# Sex-biased plasma inflammatory protein profile in obesity

**DOI:** 10.1038/s41598-026-44223-y

**Published:** 2026-03-19

**Authors:** Hilde Halland, Rui Vitorino, Eva Gerdts, Helga Midtbø, Klaus Meyer, Georgios Kararigas

**Affiliations:** 1 Center for Research on Cardiac Disease in Women, Department of Clinical Science, Bergen, Norway; 2 Department of Emergency Medicine, Haukeland University Hospital, Bergen, Norway; 3 Department of Medical Sciences, University of Aveiro, Aveiro, Portugal; 4 Department of Heart Disease, Haukeland University Hospital, Bergen, Norway; 5 Bevital, Bergen, Norway; 6 Department of Basic and Clinical Sciences, Medical School, University of Nicosia, UNIC Athens, Athens, Greece; 7 Center for Research on Cardiac Disease in Women, Department of Clinical Science, Jonas Lies vei 87, Bergen, Bergen, 5021 Norway

**Keywords:** Proteomics, Inflammation, Biological sex, Obesity., Biomarkers, Diseases, Endocrinology, Immunology, Medical research

## Abstract

Obesity-associated inflammation predisposes to cardiovascular disease (CVD). We investigated the association of biological sex with the plasma inflammatory protein profile in individuals with obesity. Clinical and proteomic data from 450 women and men with a body mass index (BMI) > 27 kg/m² without known CVD participating in the FAT associated CardiOvasculaR dysfunction study were analysed. The Olink Target 96 inflammation panel was employed in biobank samples. Hypertension was more prevalent among men, while age, BMI, and prevalences of obesity, diabetes and smoking did not differ between the sexes (all *p* > 0.05). In multivariable analyses, obesity was associated with downregulation of interleukin (IL) 7, IL 12 subunit beta, IL 6, and C-X-C motif chemokine 11, and upregulation of sulfotransferase 1A1 and SIR2-like protein 2 in women (all *p* < 0.05). In men, obesity was associated with downregulation of monocyte chemotactic protein 3, tumour necrosis factor superfamily member 12, IL 10 and protein S100-A12, and upregulation of neurotrophin-3 (all *p* < 0.05). In sum, the targeted protein profile in obesity differed by biological sex and there was no overlap in the differentially regulated proteins between women and men with obesity. The results suggest that pathophysiological mechanisms in obesity-associated inflammation and immune dysregulation may be sex-biased.

**Supplementary Information:**

The online version contains supplementary material available at 10.1038/s41598-026-44223-y.

## Introduction

The global obesity epidemic is increasing, and more than one billion people are currently living with obesity worldwide^1^. Obesity is a major driver of cardiovascular diseases (CVD) and has emerged as a particularly strong risk factor for myocardial infarction, atrial fibrillation and heart failure in women^2–7^. In clinical studies, young and middle-aged women with obesity are characterized by higher body fat mass, lower prevalence of hypertension and a higher inflammatory burden compared to men of similar age and adiposity^8–10^.

The association between obesity and chronic inflammation is well documented, both in population-based studies and in Mendelian randomisation studies^11–13^. Higher circulating levels of interleukin (IL) 6 and C-reactive protein (CRP) have been associated with both obesity and increased risk of CVD^12,14^. Conversely, anti-inflammatory agents, which reduce CRP and IL 1b, have been shown to improve CVD outcomes^15,16^. Previous studies have identified important sex-biases in obesity-associated inflammation, including larger causal estimates for the effect of adiposity on inflammatory markers^12^, and stronger associations between CVD-related biomarkers like CRP with obesity and visceral fat mass in women compared to men^13,17^. Furthermore, we recently demonstrated that having good physical fitness despite obesity was associated with a lower inflammatory burden in women, but not in men^18^.

Targeted inflammatory protein profiling through multiplexed protein detection allows efficient analysis of a vast number of proteins beyond established biomarkers and has the potential of unveiling potential mechanisms by which biological sex, obesity and inflammation interact. In turn, this may improve the current understanding of the pathophysiology of CVD in obesity. In fact, in a recent review of proteomic studies, several differentially regulated proteins (DRPs) involving multiple pathways were consistently altered in the presence of obesity^19^. In a more targeted analysis of nearly 500,000 participants in the UK Biobank, Qin et al. not only confirmed that the presence of obesity influenced the relative risk of new-onset heart failure more adversely in women than in men, but also that inflammatory pathways explained a larger proportion of obesity-associated new-onset heart failure in women than men^7^. Still, the current understanding of sex-biases in the mechanisms linking obesity and inflammation is incomplete. Thus, the present study was undertaken to further explore differences in the plasma inflammatory proteome between women and men with obesity.

## Methods

### Study population

Data from the FAT associated CardiOvasculaR dysfunction (FATCOR) study, which was conducted between 2009 and 2017 at Haukeland University Hospital, Bergen, Norway, were used^20^. From a total of 618 women and men aged 30–65 years with a body mass index (BMI) > 27.0 kg/m^2^, 450 participants (262 women and 188 men) were eligible for the present analysis (Supplementary Fig. 1). The FATCOR study was performed with approval from the Regional Committees for Medical and Health Research Ethics Western Norway (approval number REK17173) and in accordance with the Declaration of Helsinki. Written informed consent was obtained from all participants.

## Clinical assessment

All participants completed a standardised questionnaire on self-reported health, including use of any medication, smoking status and timing of last menstrual period. Obesity was recognised as BMI ≥ 30.0 kg/m^2^. Office blood pressure was measured using an Omron M4 sphygmomanometer (Omron Healthcare Co. Ltd., Hoofdorp, the Netherlands) and 24-hour ambulatory blood pressure measurement was performed using a Diasys Integra II apparatus (Novacor, Cedex, France)^20^. Hypertension was defined as elevated 24-hour ambulatory blood pressure ≥ 130/80 mmHg or use of antihypertensive medication^21^. Blood glucose, haemoglobin A_1c_ and estimated glomerular filtration rate (eGFR) were quantified in fasting, venous blood samples. Diabetes was recognised if participants reported known diabetes, or had fasting blood glucose ≥ 7 mmol/L, glycated haemoglobin A_1c_ ≥6.5% or a 2-hour blood glucose ≥ 11.1 mmol/L following an oral glucose tolerance test^22^. Follicle stimulating hormone and oestradiol were measured in serum samples using liquid chromatography with tandem mass spectrometry. Menopause was defined as more than one year since last menstruation. If the last menstrual period was unknown, women aged ≥ 53 years, who reported use of hormone replacement therapy and women with follicle stimulating hormone > 40 IU/L and oestradiol < 30 pg/mL were considered postmenopausal.

## Targeted inflammatory protein profiling

Biobank samples were drawn and stored at −80° Celsius until analysis by Bevital (www.bevital.no, Bergen, Norway). The Target 96 Inflammation panel (Olink Bioscience, Uppsala, Sweden), consisting of 92 proteins associated with inflammation and immune response, was used (https://olink.com/products/olink-target-96). The targeted protein profile was analysed in plasma using proximity extension assay technology, combining dual-recognition immunoassay and quantitative polymerase chain reaction^23^. This method provides relative quantification of protein abundance, given as normalised protein expression (NPX), Olink’s own arbitrary unit on a log_2_ scale. One unit increase in NPX indicates a doubling of protein concentration. The samples were analysed in seven batches, and the batches were intensity normalised by Olink’s Intensity normalization v.2. This adjusts the data so that median NPX for a given protein on each plate is equal to the overall median. Further, each plate was adjusted so that the median of all assays were the same for all plates. Details of the biomarker panel are given in Supplementary Table 1. Of the 92 proteins analysed, 11 proteins were excluded from the analysis, as > 50% of the samples gave results below the lower limit of detection, leaving 81 proteins for further analysis. Overall, 85% of the proteins were detected in more than 75% of the samples (Supplementary Fig. 2).

## Statistics

Data management and analyses of clinical variables were performed with IBM SPSS Statistics Version 28 (IBM, Armonk, New York, USA). Descriptive analyses were performed using Student’s t-tests for continuous variables and χ²-tests for categorical variables. Results are presented as means ± standard deviations or frequencies with percentages, as appropriate, and reported groupwise for women and men. A p-value < 0.05 was taken as statistically significant for these analyses. Biomarker analyses were performed using Jamovi (The Jamovi project 2024, version 2.5, https://www.jamovi.org). NPX data were compared between groups using permutation-based one-way ANOVA. A Benjamini–Hochberg-adjusted p-value < 0.05 was considered statistically significant to correct for multiple testing. To identify DRPs independently associated with obesity, proteins that were significantly associated with obesity in univariable analysis were further explored in multivariable linear regression models. Analyses were performed for the entire cohort, as well as stratified by sex. Models were adjusted for age, smoking status, diabetes mellitus, hypertension and eGFR. In the overall cohort, an interaction term for sex × obesity was included to test for sex-interaction, using a logistic regression model. Pearson correlation coefficients were calculated to assess associations between DRPs and relevant clinical variables within sex-specific subgroups. Proteins passing the Benjamini-Hochberg-adjusted threshold were subsequently entered into multivariable linear regression models to estimate independent associations with obesity. The p-values shown in Figs. 2 and 4 represent nominal regression p-values from these adjusted models, applied only to this pre-selected subset of proteins. We also examined the primary influence of sex (women versus men) on NPX levels in the entire population, regardless of obesity status, using multivariable linear regression adjusted for obesity, age, smoking status, diabetes mellitus, hypertension, and eGFR. The Benjamini–Hochberg correction was applied across the entire protein panel to address multiple testing. Supplementary Table 2 shows proteins associated with sex. In Fig. 5, sex and menopausal status were converted to binary variables (Sex: women = 0, men = 1; Menopause: premenopausal = 0, postmenopausal = 1).

## Results

### Clinical characteristics

**Fig. 1:**
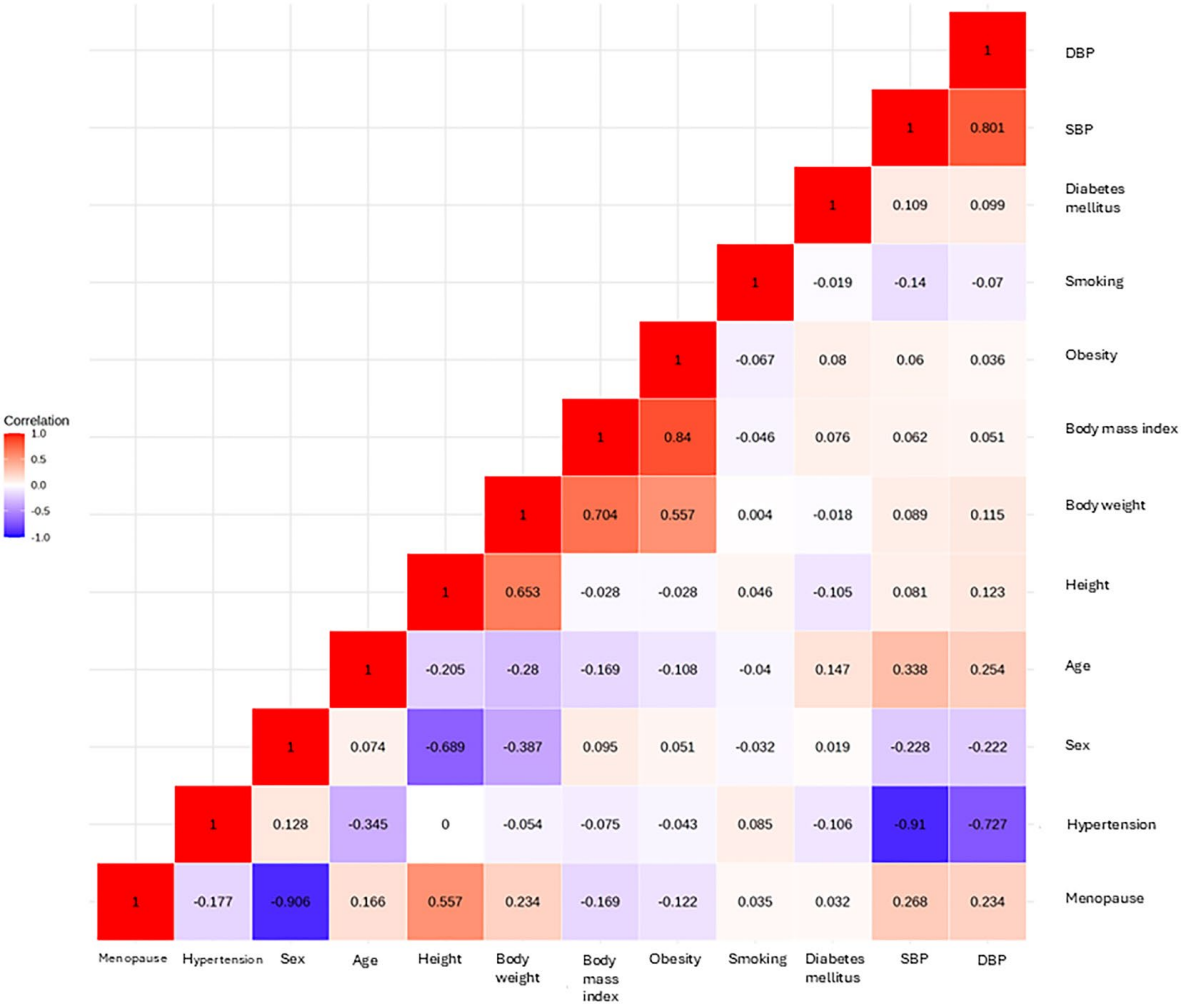
Correlation heatmap showing univariate correlation between central clinical variables, including menopause. DBP, diastolic blood pressure; SBP, systolic blood pressure. Body mass index, kg/m2. Body weight, kilograms. Height, centimetres. Age, years.

The clinical characteristics of women and men are shown in Table 1. Hypertension was more prevalent in men (74% vs. 52%, *p* < 0.001), and eGFR was lower in women (*p* < 0.05) (Table 1). Age, BMI and prevalences of obesity, diabetes and current smoking did not differ between women and men (all *p* > 0.05) (Table 1). Among women, 44% were postmenopausal (Table 1). Correlations between clinical characteristics are demonstrated in Fig. 1.

**Table 1:** Clinical characteristics of the FATCOR cohort (*n* = 450).

Covariable	Women (*n* = 262)	Men (*n* = 188)	*p*
Age, years	49 ± 9	48 ± 9	0.225
BMI, kg/m^2^	32.3 ± 4.6	31.6 ± 3.7	0.073
SBP, mmHg	127 ± 17	134 ± 14	**< 0.001**
DBP, mmHg	80 ± 9	85 ± 10	**< 0.001**
24-hour SBP, mmHg	118 ± 12	124 ± 11	**< 0.001**
24-hour DBP, mmHg	77 ± 7	81 ± 7	**< 0.001**
Fasting serum glucose, mmol/L	5.2 ± 0.8	5.5 ± 1.0	**0.001**
HbA_1c_, %	5.6 ± 0.4	5.6 ± 0.6	0.564
Estimated GFR, mL/min/1.73 m^2^	95 ± 14	97 ± 12	**0.048**
Obesity, n (%)	169 (65)	116 (62)	0.543
Diabetes mellitus, n (%)	29 (11)	20 (11)	0.885
Hypertension, n (%)	125 (52)	134 (74)	**< 0.001**
Current smoking, n (%)	32 (12)	27 (14)	0.506
Postmenopausal, n (%)	115 (44)		

BMI, body mass index; SBP, systolic blood pressure; DBP, diastolic blood pressure; HbA_1c_, haemoglobin A_1c_; GFR, glomerular filtration rate. Continuous variables were analysed using Student’s t-test and categorical variables were analysed using χ²-test. The two-tailed p-values from these analyses are shown in the table.

## Obesity and targeted protein profile in the total cohort

**Fig. 2:**
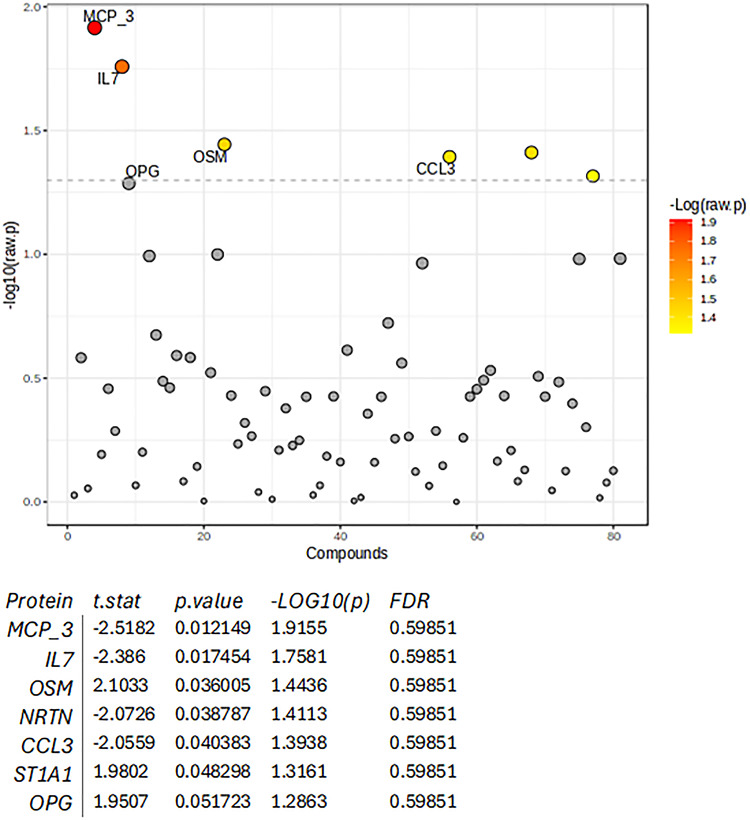
Multivariable associations between obesity and protein markers in the total study cohort. P-values are transformed by -log10 so that the more significant features with smaller p-values will be plotted higher on the graph. MCP_3, monocyte chemotactic protein 3; IL7, interleukin 7; OSM, oncostatin M; NRTN, neurturin; CCL3, C-C motif chemokine 3; ST1A1, sulfotransferase 1A1; OPG, osteoprotegerin. Only proteins significant after Benjamini–Hochberg correction in the initial screening were included. P-values shown represent nominal multivariable regression p-values and are displayed as –log10(p).

Of the 81 plasma inflammatory proteins that were analysed, seven were differentially regulated in obesity in the total cohort. In multivariable analysis, obesity was associated with upregulation of sulfotransferase 1A1, osteoprotegerin and oncostatin M, and with downregulation of monocyte chemotactic protein 3, IL 7, neurturin and C-C motif chemokine 3 in the total study cohort (Fig. 2). To test whether sex modified the relationship between obesity and the plasma inflammatory protein profile, interaction analysis was performed and did not identify any significant sex-interactions (all *p* > 0.05).

## Targeted protein profile in women and men

**Fig. 3:**
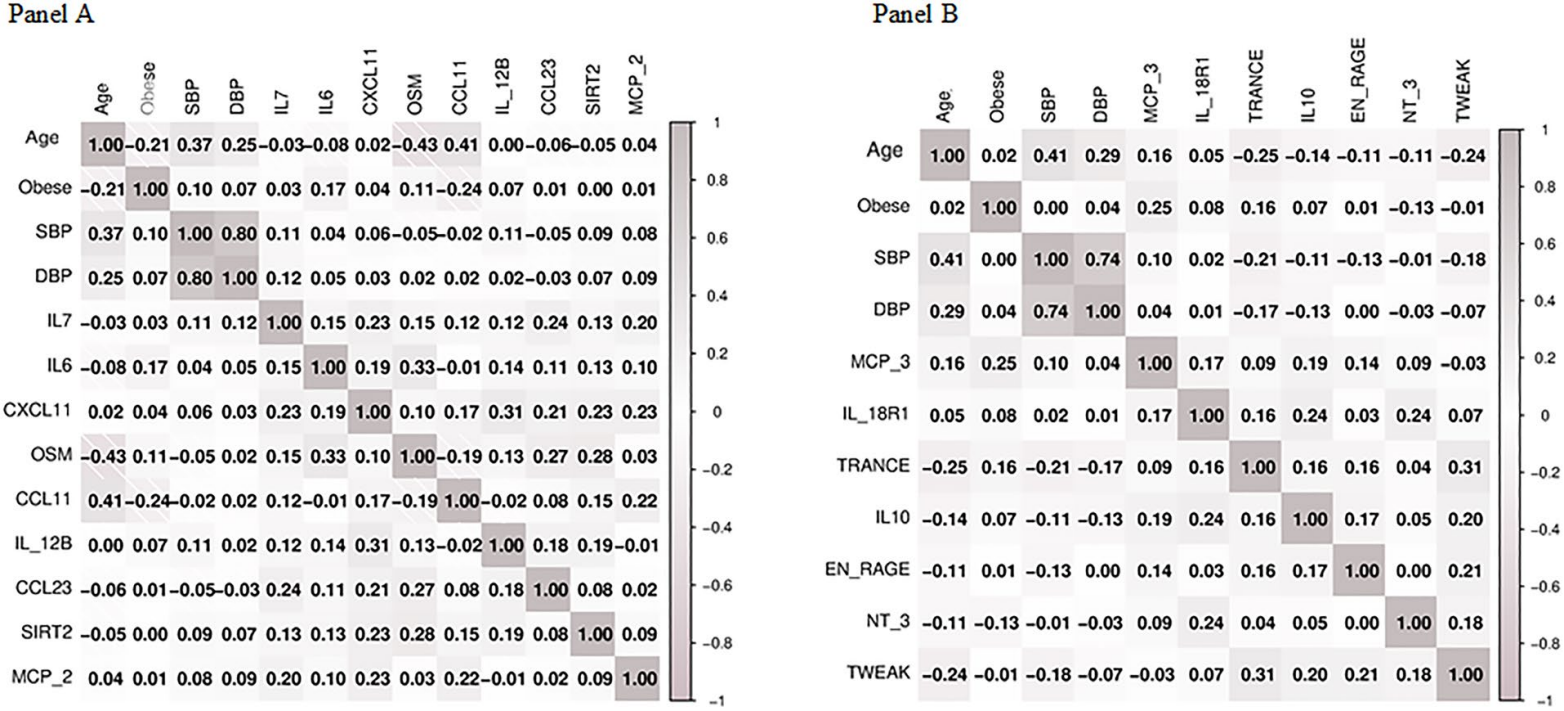
Univariate correlations between clinical variables and differentially regulated proteins in women (panel A) and men (panel B). SBP, systolic blood pressure; DBP, diastolic blood pressure; IL7, interleukin 7; IL6, interleukin 6, CXCL11, C-X-C motif chemokine 11; OSM, oncostatin M; CCL11, eotaxin; IL_12B, interleukin 12 subunit beta; CCL23, C-C motif chemokine 23; SIRT2, SIR2-like protein 2; MCP_2, monocyte chemotactic protein 2; MCP 3, monocyte chemotactic protein 3; IL_18R1, interleukin 18 receptor 1; TRANCE, TNF-related activation-induced cytokine; IL10, interleukin 10; EN_RAGE, protein S100-A12; NT_3, neurotrophin 3; TWEAK, tumour necrosis factor (ligand) superfamily member 12.

**Fig. 4:**
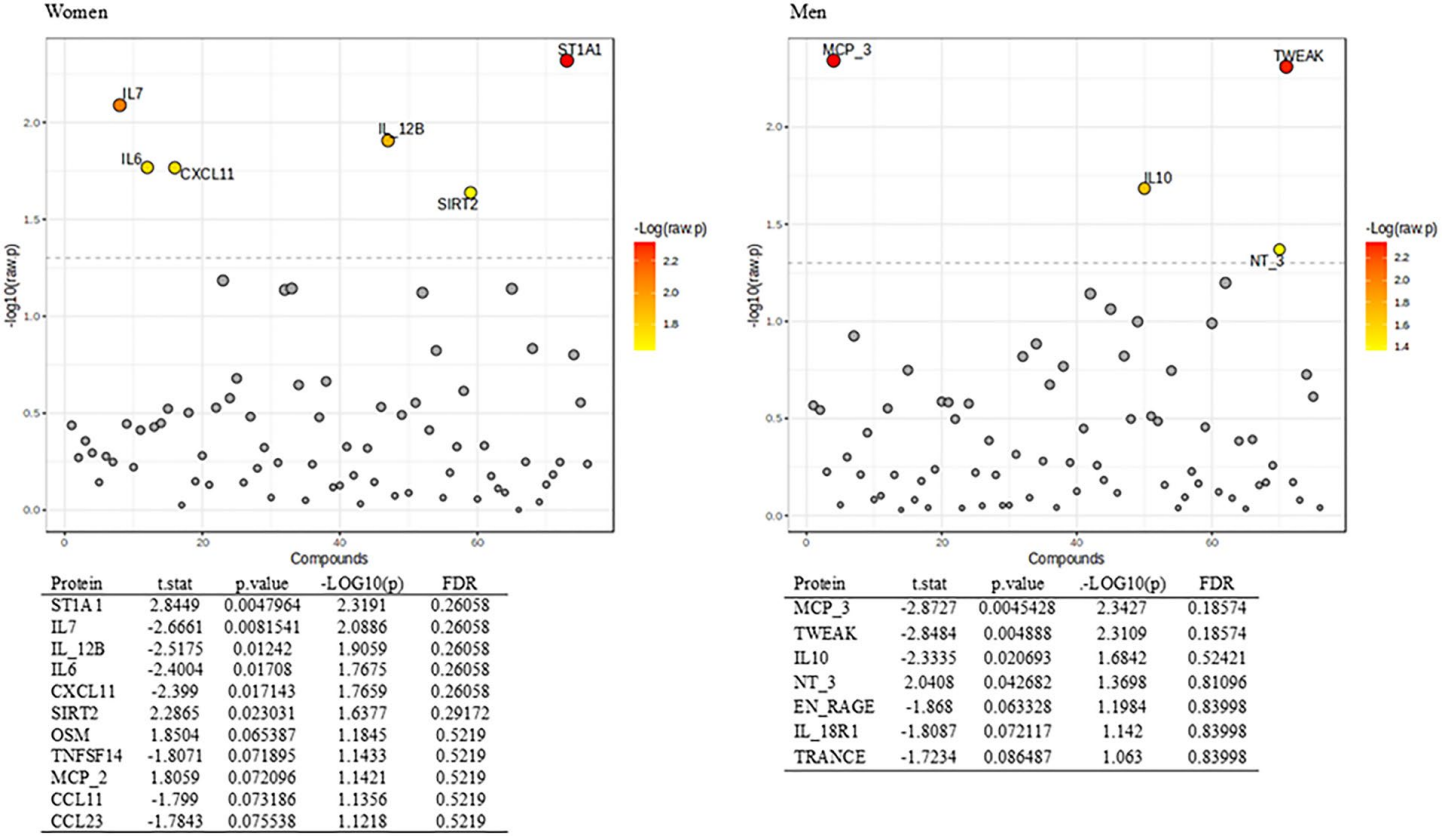
Multivariable analyses of differentially regulated proteins associated with obesity in women and men separately. P-values are transformed by -log10 so that the more significant features with smaller p-values will be plotted higher on the graph. ST1A1, sulfotransferase 1A1; IL7, interleukin 7; IL_12B, interleukin 12 subunit beta; IL6, interleukin 6; CXCL11, C-X-C motif chemokine 11; SIRT2, SIR2-like protein 2; OSM, oncostatin M; TNFSF14, tumor necrosis factor ligand superfamily 14; MCP_2, monocyte chemotactic protein 2; CCL11, eotaxin; CCL23, C-C motif chemokine 23; MCP_3, monocyte chemotactic protein 3; TWEAK, tumour necrosis factor (ligand) superfamily member 12; IL10, interleukin 10; NT_3, neurotrophin 3; EN_RAGE, protein S100-A12; IL_18R1; interleukin 18 receptor 1; TRANCE, TNF-related activation-induced cytokine. Only proteins significant after Benjamini–Hochberg correction in the initial screening were included. P-values shown represent nominal multivariable regression p-values and are displayed as –log10(p).

**Fig. 5:**
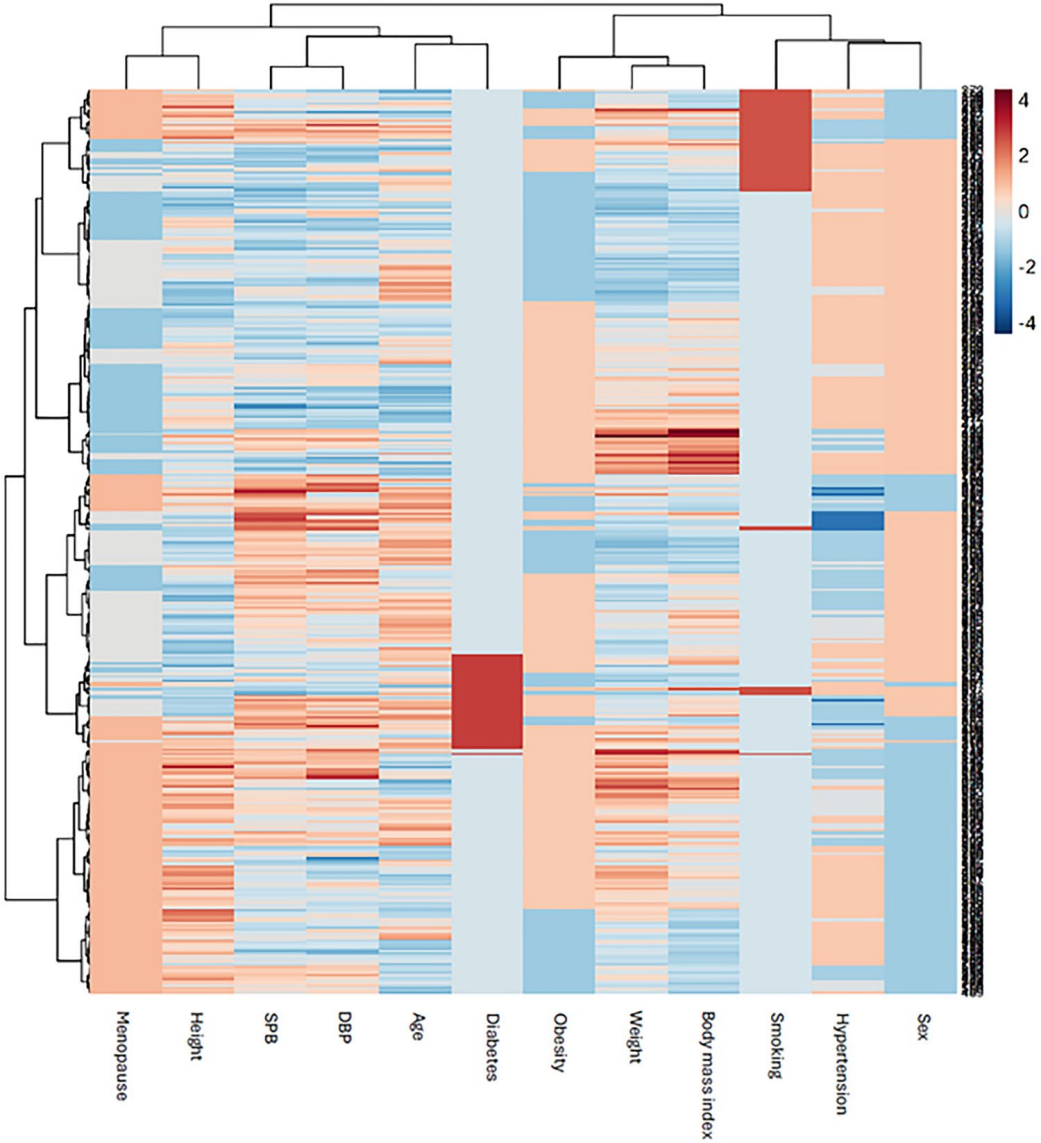
Combined molecular-clinical heatmap (total cohort). Body mass index, kg/m2. Weight, kilograms. Age, years. Height, centimetres. DBP, diastolic blood pressure; SBP, systolic blood pressure. Sex and menopausal status were encoded as binary variables for visualization (Sex: women=0, men=1; Menopause: premenopausal=0, postmenopausal=1).

Obesity was significantly correlated with IL 6, oncostatin M and C-C motif chemokine 11 in women, and with monocyte chemotactic protein-3, interleukin 10 and neurotrophin-3 in men (all *p* < 0.05) (Fig. 3). In multivariable analyses in women, presence of obesity was significantly associated with the upregulation of SIR2-like protein 2 and sulfotransferase 1A1, and with the downregulation of C-X-C chemokine 11, IL 6, IL 12 subunit beta and IL 7 (all *p* < 0.05) (Fig. 4). Menopausal status did not create a binary separation of the samples but was distributed across many molecular clusters (Fig. 5). Thus, perimenopausal transition did not produce a consistent proteomic signature change in women in the present cohort (Fig. 5).

In multivariable analyses in men, obesity was significantly associated with the upregulation of neurotrophin-3, and with the downregulation of monocyte chemotactic protein 3, tumour necrosis factor superfamily ligand member 12, IL 10 and protein S100A12 in multivariable analyses (all *p* < 0.05) (Fig. 4). Notably, despite the lack of a significant sex-interaction test in the previous analysis, the DRPs associated with obesity showed no overlap between women and men (Fig. 4). In multivariable models adjusted for obesity and clinical covariates, several inflammatory proteins differed significantly between women and men after Benjamini–Hochberg correction (Supplementary Table 2), indicating sex-associated differences in circulating inflammatory protein abundance that are not explained solely by obesity status.

## Discussion

This exploratory study adds to current knowledge by demonstrating that the plasma inflammatory targeted protein profile differs significantly between women and men with obesity. Overall, the pattern of DRPs associated with obesity point to a complex interaction between several pathways including, but not limited to, hormone metabolism, liver function, neuronal maintenance, immune cell recruitment and several other aspects of inflammation. Notably, the lack of overlap between the DRPs significantly associated with obesity in women and men, respectively, may indicate that different pathways are engaged in the development and maintenance of obesity-associated inflammation and immune dysfunction between women and men.

Certain proteins that differ between women and men, regardless of adiposity, are regulated by sex hormones and participate in immunological pathways with established sexual-dependent regulation. Oestrogens primarily enhance humoral immunity while reducing pro-inflammatory myeloid responses, partly through oestrogen receptor-mediated inhibition of NF-κB activation and regulation of cytokine transcription. In contrast, androgens generally exert immunosuppressive effects^24^. In line with this, we observed significant sex differences in chemokines involved in leukocyte recruitment, survival mechanisms, and growth factor-related signalling. Oestrogen signalling influences the expression of IL-6 family cytokines and chemokines, while androgen signalling affects monocyte and macrophage activity and TNF-related pathways^25^. These mechanisms may account for the higher prevalence of specific inflammatory and immune-regulatory proteins in women and the comparatively lower levels of anti-inflammatory mediators, particularly IL-10–related pathways, in men. These findings suggest that intrinsic hormonal modulation of immune signalling shapes the circulating inflammatory protein profile, providing a biological basis for sex-biased cardiometabolic risk in obesity.

Several of the inflammatory proteins mentioned above have also been associated with CVD, enabling differentiation between inflammatory drivers common to both sexes and those unique to one sex. Proteins, such as osteoprotegerin and oncostatin M, which were linked to obesity in the overall group, have been associated with vascular remodelling, cardiac stress, and adverse cardiovascular outcomes^26^. This indicates that both men and women share inflammatory pathways that increase risk of CVD. However, sex-stratified analysis revealed largely distinct protein signatures, suggesting unique upstream processes. In women, alterations in IL-6 family cytokines and chemokines that regulate immune cell trafficking and adaptive responses may promote persistent low-grade inflammation and endothelial dysfunction. In men, obesity-induced changes in neurotrophin-3, TNF-related signalling, and reduced IL-10-mediated anti-inflammatory activity may impair inflammatory resolution and worsen adverse metabolic-vascular interactions. Collectively, these data support the concept that obesity-related cardiovascular risk arises from shared inflammatory mediators superimposed on sex-specific immunological and reparative mechanisms. This divergence may explain observed sex differences in cardiometabolic phenotypes and suggests that future preventive or therapeutic strategies could benefit from sex-dependent targeting of inflammatory pathways.

In the current study, there was an increased abundance of SIR2-like protein 2 and sulfotransferase 1A1 in women. Previous studies have found that SIR2-like protein 2 is involved in multiple pathways in cell differentiation and inflammation^27–30^, and that it in a context-dependent fashion may relay both beneficial and harmful responses in different CVDs^31^. Sulfotransferase 1A1 has been suggested to have multiple and important roles in hormone metabolism and liver function^32^, and is proposed to indirectly influence susceptibility to CVD^33^. Furthermore, obesity was associated with reduced abundance of C-X-C chemokine 11, IL 6, IL 12 subunit beta and IL 7 in women. Separately and together, these cytokines are linked to development and regulation of immune cells^32,34–37^, and may also contribute to several aspects of inflammatory responses, some implicated in CVD pathogenesis^38–40^. The leptin/adiponectin-ratio, an indicator of metabolic health, has previously been associated with hypertension in women^41,42^. Among the DRPs identified in women in the present cohort, only IL 6 is likely to interact directly with leptin and adiponectin^43^. However, these adipokines were not measured in the present study, and the significance of the observed reduced abundance of IL 6 must be assessed in larger protein panels or in untargeted studies including assessment of pathways. Taken together, it is possible that the identified DRPs may contribute to the more pronounced chronic inflammation and immune dysfunction observed in women with obesity^10,13^. It should also be kept in mind that circulating concentrations of inflammatory proteins may not accurately represent local tissue-level inflammation or underlying regulatory mechanisms, such as epigenetic activation.

In men, the presence of obesity was associated with a significantly higher abundance of neurotrophin-3, which is best known for its role in neuronal regulation^44^. However, neurotrophin-3 also is likely involved in regulating survival and function of cells in the CV system, and may be involved in atherogenesis, vascular remodelling and inflammation^45^. Significantly lower abundances of monocyte chemotactic protein 3, involved in recruitment of lymphocytes^46^, tumour necrosis factor superfamily ligand member 12, involved in apoptosis and adipose tissue dynamics^47^, IL 10, an anti-inflammatory cytokine^46^, and protein S100A12, involved in cytokine production and oxidative stress^48^, were found in men. Taken together, these findings may indicate that men with obesity potentially are subject to several important alterations, including changes in cell survival and signalling in inflammation. In addition, the reduced abundance of IL 10 may indicate reduced ability to balance inflammation, which may worsen metabolic dysfunction^49^. This finding is of particular interest in light of previous publications showing a positive association between IL 10 and insulin sensitivity^50^, a reduced abundance of IL 10 in cases of newly diagnosed type 2 diabetes^51^, and that men develop type 2 diabetes at a lower BMI than women^52^.

In line with the finding of different circulating inflammatory protein profiles between women and men in the present study, also the Dallas Heart study demonstrated that protein biomarkers relevant for CVD differed significantly between women and men without known CVD^53^. Although the biomarker panels in the Dallas Heart Study and the present cohort differed, both studies found upregulation of osteoprotegerin. Furthermore, both studies identified sex differences in protein biomarkers that previously have been associated with obesity, endothelial dysfunction, inflammatory cell recruitment and cardiac stress/injury^53^. The present study adds to those findings and advances the field by reporting sex-biased results from a biomarker panel more focused on immune function and inflammation.

In the total FATCOR cohort, obesity was significantly associated with increased abundance of osteoprotegerin and oncostatin M and lower abundance of CC-motif chemokine 3. Osteoprotegerin is known for involvement in bone metabolism, atherogenesis and vascular calcification, and in a meta-analysis, higher levels of osteoprotegerin was associated with increased CVD risk^54^. Oncostatin M, a member of the IL 6 cytokine family, is involved in metabolism, inflammation and tissue regeneration, and may contribute to the systemic inflammation observed in obesity^55^. CC-motif chemokine 3 is involved in inflammation and may contribute to the altered immune function in obesity^56^.

Obesity was also associated with reduced abundance of monocyte chemotactic protein 3 in men and CC-motif chemokine 3 in the total cohort. In previous studies both of these proteins have been shown to be involved in cell recruitment^46,56^. This contrasts the findings from China Kadoorie Biobank^11^. In that study, higher BMI was associated with increased abundance of these proteins both in observational and Mendelian randomisation analyses^11^. However, the Chinese cohort mainly included normal weight individuals and only 4% had a BMI > 30 kg/m^2 11^. The divergent results may reflect differences in body composition between the cohorts. However, both the present study and the Chinese cohort relied on BMI as the sole indicator of obesity. It is possible that analyses using other measures of adiposity more sensitive to differences in body composition would improve the understanding of the findings.

The Gutenberg Health Study performed in 6662 individuals, including participants with known CVD, recently reported an association of an obesity-related inflammatory protein signature with risk of all-cause mortality, cardiac death, incident coronary artery disease and a major adverse CV event endpoint consisting of nonfatal stroke, nonfatal myocardial infarction and CV death^57^. In particular, obesity was associated with increased abundance of neurotrophin-3 and reduced abundance of tumour necrosis factor ligand superfamily 12 ^57^, as also found in men in the present study.

Finally, in a study of almost 40,000 participants in the UK Biobank, Royer et al. investigated whether prediction of major CV events was improved by adding data from large-scale proteomics to established CV risk factors^58^. Although several potentially relevant proteins were identified, the overall impact on risk prediction was small. Despite not providing results stratified by sex, an immunological pathway involving IL 12 subunit beta was enriched, as also identified in women in the present study^58^.

### Study limitations

The FATCOR study predominantly included Caucasians living in Western Norway, and extrapolation of the results to other more ethnically and metabolically diverse cohorts should be done with caution. As the results stem from a single cohort in Norway, confirmation of the results should be sought in larger, multi-center cohorts, with particular attention to sex-dependent interactions and mechanistic validation. Causal and temporal relationships between obesity, sex, biomarkers and clinical covariables could not be tested due to the cross-sectional design of the FATCOR study. Also, the study does not include a normal weight group for comparison, and no participants with BMI 25–27 kg/m^2^ were included. Any subtle changes that may have occurred within this BMI-range will therefore not have been detected. Furthermore, the duration of obesity at the time of the study visit was not known. The protein biomarkers were measured only once, and as such markers can vary over time, introduction of a bias cannot be ruled out. Furthermore, by analysing only one panel of inflammatory proteins, there is a possibility of being blinded to relevant associations with other types of proteins. However, we chose a well-suited inflammatory protein panel for this investigation, and the samples were handled as recommended in the preanalytical phase and analysed by a single, accredited laboratory in close proximity to sample acquisition and storage. Furthermore, a hypothesis-free approach was used in the biomarker analysis, reducing the risk of overlooking important relationships. Given the experimental design, the current study was exploratory and untargeted in nature, and relationships between proteins and pathways was therefore not expanded on. In future untargeted studies, the exploration of such relationships and pathway enrichment analysis could provide a broader understanding.

## Conclusions

This study expands current knowledge by demonstrating sex-biases in the circulating plasma inflammatory proteome profile in obesity. Despite absence of a significant interaction with sex, here was no overlap in the DRPs found in women and men, suggesting that different pathophysiological mechanisms may be driving the obesity-associated inflammation and immune dysregulation between women and men. Confirmation of the results should be sought in future research, with particular attention to sex-dependent interactions and mechanistic validation. In obese subjects without known CVD, understanding the role of sex is of utmost importance to identify future targets for improved CVD prevention in women and men.

## Supplementary Information

Below is the link to the electronic supplementary material.

Supplementary Material 1

## Data Availability

Upon reasonable request to the corresponding author, the data from the current study may be made available.

## References

[1] Koskinas, K. C. et al. Obesity and cardiovascular disease: an ESC clinical consensus statement. *Eur. Heart J.* **45**, 4063–4098. 10.1093/eurheartj/ehae508 (2024). 39210706 10.1093/eurheartj/ehae508

[2] van Essen, B. J. et al. Sex-specific risk factors for new-onset heart failure: the PREVEND study at 25 years. *Eur. Heart J.* **46**, 1528–1536. 10.1093/eurheartj/ehae868 (2025). 39786471 10.1093/eurheartj/ehae868 PMC12011521

[3] Manrique-Acevedo, C., Chinnakotla, B., Padilla, J., Martinez-Lemus, L. A. & Gozal, D. Obesity and cardiovascular disease in women. *Int. J. Obes. (Lond)*. **44**, 1210–1226. 10.1038/s41366-020-0548-0 (2020). 32066824 10.1038/s41366-020-0548-0 PMC7478041

[4] Collaborators, G. B. D. O. et al. Health Effects of Overweight and Obesity in 195 Countries over 25 Years. *N Engl. J. Med.* **377**, 13–27. 10.1056/NEJMoa1614362 (2017). 28604169 10.1056/NEJMoa1614362 PMC5477817

[5] Proietti, M., Raparelli, V., Basili, S., Olshansky, B. & Lip, G. Y. Relation of female sex to left atrial diameter and cardiovascular death in atrial fibrillation: The AFFIRM Trial. *Int. J. Cardiol.* **207**, 258–263. 10.1016/j.ijcard.2016.01.169 (2016). 26808988 10.1016/j.ijcard.2016.01.169

[6] Savji, N. et al. The Association of Obesity and Cardiometabolic Traits With Incident HFpEF and HFrEF. *JACC Heart Fail.* **6**, 701–709. 10.1016/j.jchf.2018.05.018 (2018). 30007554 10.1016/j.jchf.2018.05.018 PMC6076337

[7] Qin, H. et al. Clinical and Proteomic Risk Profiles of New-Onset Heart Failure in Men and Women. *JACC Heart Fail.*10.1016/j.jchf.2024.09.022 (2024). 39708029 10.1016/j.jchf.2024.09.022

[8] Chen, Z. et al. China Kadoorie Biobank of 0.5 million people: survey methods, baseline characteristics and long-term follow-up. *Int. J. Epidemiol.* **40**, 1652–1666. 10.1093/ije/dyr120 (2011). 22158673 10.1093/ije/dyr120 PMC3235021

[9] Halland, H. et al. Sex differences in subclinical cardiac disease in overweight and obesity (the FATCOR study). *Nutr. Metab. Cardiovasc. Dis.* **28**, 1054–1060. 10.1016/j.numecd.2018.06.014 (2018). 30177273 10.1016/j.numecd.2018.06.014

[10] Liqiang, S., Fang-Hui, L., Minghui, Q. & Haichun, C. Threshold effect and sex characteristics of the relationship between chronic inflammation and BMI. *BMC Endocr. Disord*. **23**, 175. 10.1186/s12902-023-01396-1 (2023). 37582770 10.1186/s12902-023-01396-1 PMC10428651

[11] Pang, Y. et al. Associations of Adiposity, Circulating Protein Biomarkers, and Risk of Major Vascular Diseases. *JAMA Cardiol.* **6**, 276–286. 10.1001/jamacardio.2020.6041 (2021). 33263724 10.1001/jamacardio.2020.6041 PMC7711564

[12] Fall, T. et al. Age- and sex-specific causal effects of adiposity on cardiovascular risk factors. *Diabetes* **64**, 1841–1852. 10.2337/db14-0988 (2015). 25712996 10.2337/db14-0988 PMC4407863

[13] Choi, J., Joseph, L. & Pilote, L. Obesity and C-reactive protein in various populations: a systematic review and meta-analysis. *Obes. Rev.* **14**, 232–244. 10.1111/obr.12003 (2013). 23171381 10.1111/obr.12003

[14] Kaptoge, S. et al. Inflammatory cytokines and risk of coronary heart disease: new prospective study and updated meta-analysis. *Eur. Heart J.* **35**, 578–589. 10.1093/eurheartj/eht367 (2014). 24026779 10.1093/eurheartj/eht367 PMC3938862

[15] Ridker, P. M. et al. Antiinflammatory Therapy with Canakinumab for Atherosclerotic Disease. *N Engl. J. Med.* **377**, 1119–1131. 10.1056/NEJMoa1707914 (2017). 28845751 10.1056/NEJMoa1707914

[16] Tardif, J. C. et al. Efficacy and Safety of Low-Dose Colchicine after Myocardial Infarction. *N Engl. J. Med.* **381**, 2497–2505. 10.1056/NEJMoa1912388 (2019). 31733140 10.1056/NEJMoa1912388

[17] Ramirez, M. F. et al. Sex Differences in Protein Biomarkers and Measures of Fat Distribution. *J. Am. Heart Assoc.* **13**, e000223. 10.1161/JAHA.124.000223 (2024). 39526334 10.1161/JAHA.124.000223 PMC11681412

[18] Midtbo, H. et al. Influence of cardiorespiratory fitness on obesity-associated inflammation in women and men: The FATCOR study. *Nutr. Metab. Cardiovasc. Dis.* **34**, 1942–1949. 10.1016/j.numecd.2024.04.002 (2024). 38749786 10.1016/j.numecd.2024.04.002

[19] Rodriguez-Munoz, A. et al. A Systematic Review of Proteomics in Obesity: Unpacking the Molecular Puzzle. *Curr. Obes. Rep.*10.1007/s13679-024-00561-4 (2024). 38703299 10.1007/s13679-024-00561-4 PMC11306592

[20] Eikas, J. G. et al. Arterial Stiffness in Overweight and Obesity: Association with Sex, Age, and Blood Pressure. *High. Blood Press. Cardiovasc. Prev.* **30**, 435–443. 10.1007/s40292-023-00593-2 (2023). 37505440 10.1007/s40292-023-00593-2 PMC10600283

[21] Mancia, G. et al. ESH Guidelines for the management of arterial hypertension The Task Force for the management of arterial hypertension of the European Society of Hypertension: Endorsed by the International Society of Hypertension (ISH) and the European Renal Association (ERA). *J Hypertens* 41, 1874–2071, (2023). 10.1097/HJH.0000000000003480 (2023). 10.1097/HJH.0000000000003480 37345492

[22] American Diabetes, A. Diagnosis and classification of diabetes mellitus. *Diabetes Care*. **37** (Suppl 1), 81–90. 10.2337/dc14-S081 (2014).23959568

[23] Assarsson, E. et al. Homogenous 96-plex PEA immunoassay exhibiting high sensitivity, specificity, and excellent scalability. *PLoS One*. **9**, e95192. 10.1371/journal.pone.0095192 (2014). 24755770 10.1371/journal.pone.0095192 PMC3995906

[24] Simoes, E. et al. Sex dimorphism in inflammatory response to obesity in childhood. *Int. J. Obes.* **45**, 879–887. 10.1038/s41366-021-00753-1 (2021). 10.1038/s41366-021-00753-1 PMC8005372 33526854

[25] Harding, A. T. & Heaton, N. S. The Impact of Estrogens and Their Receptors on Immunity and Inflammation during Infection. *Cancers* **14**10.3390/cancers14040909 (2022). 10.3390/cancers14040909 PMC8870346 35205657

[26] Pérez de Ciriza, C., Lawrie, A. & Varo, N. Osteoprotegerin in Cardiometabolic Disorders. *Int. J. Endocrinol.* **2015** (564934). 10.1155/2015/564934 (2015). 10.1155/2015/564934 PMC4442310 26078757

[27] Rodriguez, R. M., Fernandez, A. F. & Fraga, M. F. Role of sirtuins in stem cell differentiation. *Genes Cancer*. **4**, 105–111. 10.1177/1947601913479798 (2013). 24020001 10.1177/1947601913479798 PMC3764466

[28] Tao, Z., Jin, Z., Wu, J., Cai, G. & Yu, X. Sirtuin family in autoimmune diseases. *Front. Immunol.* **14**, 1186231. 10.3389/fimmu.2023.1186231 (2023). 37483618 10.3389/fimmu.2023.1186231 PMC10357840

[29] Wu, Q. J. et al. The sirtuin family in health and disease. *Signal. Transduct. Target. Ther.* **7**, 402. 10.1038/s41392-022-01257-8 (2022). 36581622 10.1038/s41392-022-01257-8 PMC9797940

[30] Sola-Sevilla, N., Garmendia-Berges, M., Mera-Delgado, M. & Puerta, E. Context-dependent role of sirtuin 2 in inflammation. *Neural Regen Res.* **20**, 682–694. 10.4103/NRR.NRR-D-23-02063 (2025). 38886935 10.4103/NRR.NRR-D-23-02063 PMC11433891

[31] Alhasaniah, A. H. et al. The enigmatic role of SIRT2 in the cardiovascular system: Deciphering its protective and detrimental actions to unlock new avenues for therapeutic intervention. *Curr. Probl. Cardiol.* **50**, 102929. 10.1016/j.cpcardiol.2024.102929 (2025). 39566866 10.1016/j.cpcardiol.2024.102929

[32] Tabrett, C. A. & Coughtrie, M. W. Phenol sulfotransferase 1A1 activity in human liver: kinetic properties, interindividual variation and re-evaluation of the suitability of 4-nitrophenol as a probe substrate. *Biochem. Pharmacol.* **66**, 2089–2097. 10.1016/s0006-2952(03)00582-3 (2003). 14609733 10.1016/s0006-2952(03)00582-3

[33] O’Halloran, A. M. et al. Genetic polymorphisms in platelet-related proteins and coronary artery disease: investigation of candidate genes, including N-acetylgalactosaminyltransferase 4 (GALNT4) and sulphotransferase 1A1/2 (SULT1A1/2). *J. Thromb. Thrombolysis*. **27**, 175–184. 10.1007/s11239-008-0196-z (2009). 18259693 10.1007/s11239-008-0196-z

[34] Wang, J., Ouyang, X., Zhu, W., Yi, Q. & Zhong, J. The Role of CXCL11 and its Receptors in Cancer: Prospective but Challenging Clinical Targets. *Cancer Control*. **31**, 10732748241241162. 10.1177/10732748241241162 (2024). 38533911 10.1177/10732748241241162 PMC10976495

[35] Rose-John, S., Jenkins, B. J., Garbers, C., Moll, J. M. & Scheller, J. Targeting IL-6 trans-signalling: past, present and future prospects. *Nat. Rev. Immunol.* **23**, 666–681. 10.1038/s41577-023-00856-y (2023). 37069261 10.1038/s41577-023-00856-y PMC10108826

[36] Trinchieri, G. Interleukin-12 and the regulation of innate resistance and adaptive immunity. *Nat. Rev. Immunol.* **3**, 133–146. 10.1038/nri1001 (2003). 12563297 10.1038/nri1001

[37] Chen, D., Tang, T. X., Deng, H., Yang, X. P. & Tang, Z. H. Interleukin-7 Biology and Its Effects on Immune Cells: Mediator of Generation, Differentiation, Survival, and Homeostasis. *Front. Immunol.* **12**, 747324. 10.3389/fimmu.2021.747324 (2021). 34925323 10.3389/fimmu.2021.747324 PMC8674869

[38] Lu, X. et al. The Role of CXC Chemokines in Cardiovascular Diseases. *Front. Pharmacol.* **12**, 765768. 10.3389/fphar.2021.765768 (2021). 35668739 10.3389/fphar.2021.765768 PMC9163960

[39] Li, R. et al. Interleukin-7 induces recruitment of monocytes/macrophages to endothelium. *Eur. Heart J.* **33**, 3114–3123. 10.1093/eurheartj/ehr245 (2012). 21804111 10.1093/eurheartj/ehr245 PMC3598429

[40] Posadas-Sanchez, R. & Vargas-Alarcon, G. Innate Immunity in Coronary Disease. The Role of Interleukin-12 Cytokine Family in Atherosclerosis. *Rev. Invest. Clin.* **70**, 5–17. 10.24875/RIC.17002335 (2018). 29513302 10.24875/RIC.17002335

[41] Faulkner, J. L. & de Belin, E. J. Sex Differences in Mechanisms of Hypertension Associated With Obesity. *Hypertension* **71**, 15–21. 10.1161/HYPERTENSIONAHA.117.09980 (2018). 29133358 10.1161/HYPERTENSIONAHA.117.09980 PMC5730468

[42] Hall, J. E., Carmo, da Silva, J. M., Wang, A. A., Hall, M. E. & Z. & Obesity-induced hypertension: interaction of neurohumoral and renal mechanisms. *Circ. Res.* **116**, 991–1006. 10.1161/CIRCRESAHA.116.305697 (2015). 25767285 10.1161/CIRCRESAHA.116.305697 PMC4363087

[43] Ter Horst, R. et al. Sex-Specific Regulation of Inflammation and Metabolic Syndrome in Obesity. *Arterioscler. Thromb. Vasc Biol.* **40**, 1787–1800. 10.1161/ATVBAHA.120.314508 (2020). 32460579 10.1161/ATVBAHA.120.314508 PMC7310302

[44] Vilar, M. & Mira, H. Regulation of Neurogenesis by Neurotrophins during Adulthood: Expected and Unexpected Roles. *Front. Neurosci.* **10**, 26. 10.3389/fnins.2016.00026 (2016). 26903794 10.3389/fnins.2016.00026 PMC4746328

[45] Caporali, A. & Emanueli, C. Cardiovascular actions of neurotrophins. *Physiol. Rev.* **89**, 279–308. 10.1152/physrev.00007.2008 (2009). 19126759 10.1152/physrev.00007.2008 PMC2836529

[46] Rollins, B. J. *Chemokines Blood* **90**, 909–928 (1997). 9242519

[47] Shunkina Skuratovskaia, D. et al. Tumor Necrosis Receptor Superfamily Interact with Fusion and Fission of Mitochondria of Adipose Tissue in Obese Patients without Type 2 Diabetes. *Biomedicines* **9**10.3390/biomedicines9091260 (2021). 10.3390/biomedicines9091260 PMC8470627 34572446

[48] Meijer, B., Gearry, R. B. & Day, A. S. The role of S100A12 as a systemic marker of inflammation. *Int J Inflam* 907078, (2012). 10.1155/2012/907078 (2012). 10.1155/2012/907078 PMC3395136 22811950

[49] Saraiva, M., Vieira, P. & O’Garra, A. Biology and therapeutic potential of interleukin-10. *J. Exp. Med.* **217**10.1084/jem.20190418 (2020). 10.1084/jem.20190418 PMC7037253 31611251

[50] Straczkowski, M., Kowalska, I., Nikolajuk, A., Krukowska, A. & Gorska, M. Plasma interleukin-10 concentration is positively related to insulin sensitivity in young healthy individuals. *Diabetes Care*. **28**, 2036–2037. 10.2337/diacare.28.8.2036 (2005). 16043753 10.2337/diacare.28.8.2036

[51] Abhilasha et al. Downregulation of interleukin-10 receptor (IL-10R) along with low serum IL-10 levels in newly diagnosed type 2 diabetes mellitus patients. *Gene Rep.* **24**10.1016/j.genrep.2021.101251 (2021).

[52] Logue, J. et al. Do men develop type 2 diabetes at lower body mass indices than women? *Diabetologia* **54**, 3003–3006. 10.1007/s00125-011-2313-3 (2011). 21959958 10.1007/s00125-011-2313-3 PMC4220585

[53] Lew, J. et al. Sex-Based Differences in Cardiometabolic Biomarkers. *Circulation* **135**, 544–555. 10.1161/CIRCULATIONAHA.116.023005 (2017). 28153991 10.1161/CIRCULATIONAHA.116.023005 PMC5302552

[54] Tschiderer, L. et al. Osteoprotegerin and Cardiovascular Events in High-Risk Populations: Meta-Analysis of 19 Prospective Studies Involving 27 450 Participants. *J. Am. Heart Assoc.* **7**, e009012. 10.1161/JAHA.118.009012 (2018). 30369329 10.1161/JAHA.118.009012 PMC6201389

[55] Jones, S. A. & Jenkins, B. J. Recent insights into targeting the IL-6 cytokine family in inflammatory diseases and cancer. *Nat. Rev. Immunol.* **18**, 773–789. 10.1038/s41577-018-0066-7 (2018). 30254251 10.1038/s41577-018-0066-7

[56] He, W., Wang, H., Yang, G., Zhu, L. & Liu, X. The Role of Chemokines in Obesity and Exercise-Induced Weight Loss. *Biomolecules* 14, (2024). 10.3390/biom14091121 10.3390/biom14091121 PMC11430256 39334887

[57] Panova-Noeva, M. et al. Obesity-related inflammatory protein signature in cardiovascular clinical outcomes: results from the Gutenberg Health Study. *Obes. (Silver Spring)*. **32**, 1198–1209. 10.1002/oby.24014 (2024). 10.1002/oby.24014 38664310

[58] Royer, P. et al. Large-scale plasma proteomics in the UK Biobank modestly improves prediction of major cardiovascular events in a population without previous cardiovascular disease. *Eur. J. Prev. Cardiol.* **31**, 1681–1689. 10.1093/eurjpc/zwae124 (2024). 38546334 10.1093/eurjpc/zwae124

