# Supplementary material for: Sex-biased plasma inflammatory protein profile in obesity
Source: Sci Rep. 2026 Mar 19;16:14249. doi: 10.1038/s41598-026-44223-y (PMC13139431; doi:10.1038/s41598-026-44223-y)
Supplement: Supplementary file 1 — Supplementary Material 1 [file 41598_2026_44223_MOESM1_ESM.pdf]

Supplementary information

Sex-biased plasma inflammatory protein profile in obesity

Hilde Halland[1,2*], Rui Vitorino[3], Eva Gerdts[1,4], Helga Midtbø[1,4], Klaus Meyer[5], Georgios Kararigas[6].

Supplementary figure 1

Overview of participant inclusion and exclusion for the current analysis.

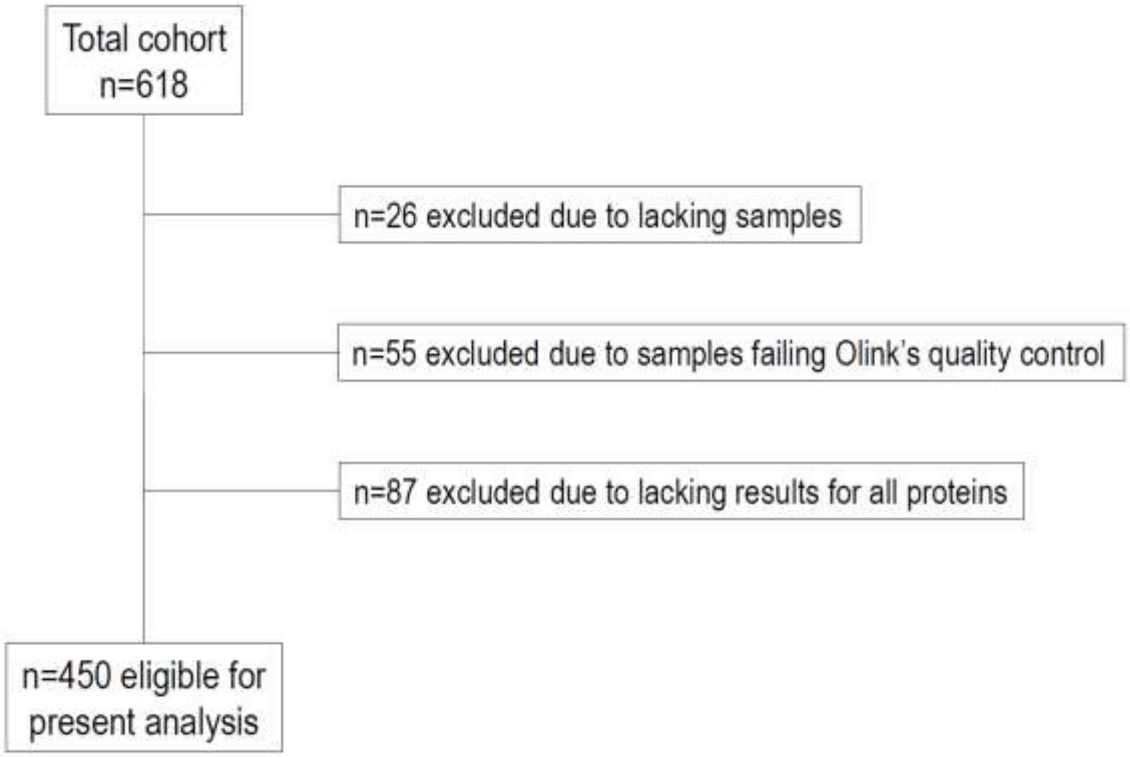

Supplementary Figure 2

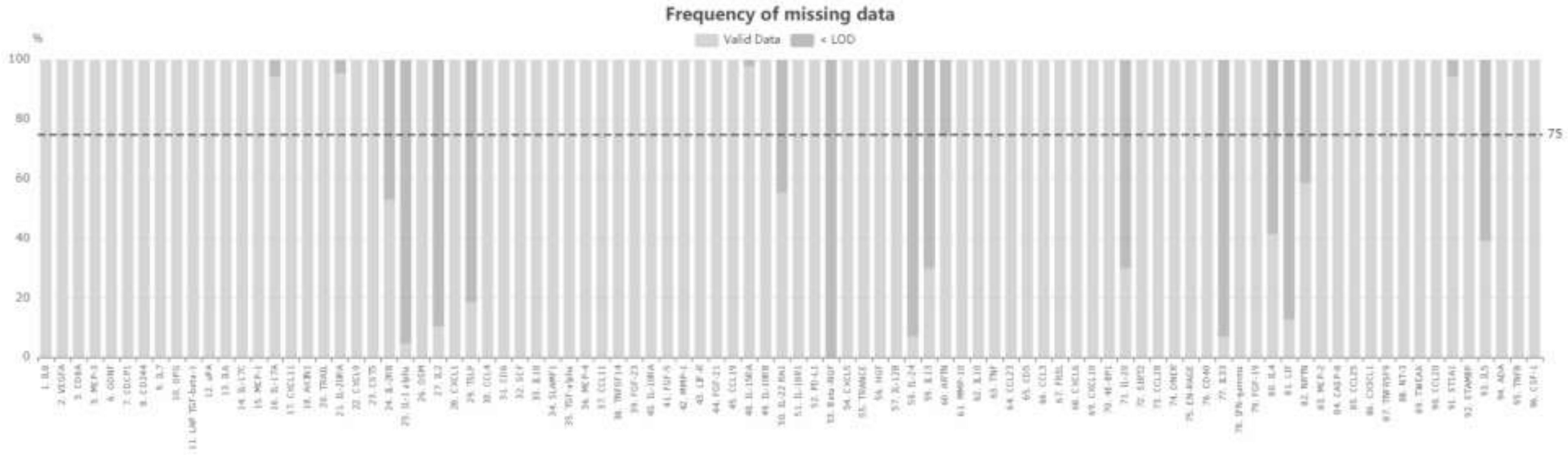

Bar chart plot showing protein detectability as relative number of samples above or below limit of detection.

Two samples failure during analyses (0.34%) and are not shown in the plot. The dotted line shows the expected detectability for the Olink inflammation panel (85%, based on EDTA plasma from healthy donors. LOD; limit of detection.

Supplementary table 1

Overview of proteins included in the Olink Target 96 inflammation panel.

Abbreviation	Name	Abbreviation	Name
ADA	Adenosine Deaminase	Flt3L	Fms-related tyrosine kinase 3 ligand
ARTN	Artemin	CX3CL1	Fractalkine
AXIN1	Axin-1	GDNF	Glial cell line-derived neurotrophic factor
Beta-NGF	Beta-nerve growth factor	HGF	Hepatocyte growth factor
CASP-8	Caspase-8	IFN-gamma	Interferon gamma
CCL3	C-C motif chemokine 3	IL-1 alpha	Interleukin-1-alpha
CCL4	C-C motif chemokine 4	IL-2	Interleukin-2
CCL19	C-C motif chemokine 19	IL-2RB	Interleukin-2 receptor subunit beta
CCL20	C-C motif chemokine 20	IL-4	Interleukin-4
CCL23	C-C motif chemokine 23	IL-5	Interleukin-5
CCL25	C-C motif chemokine 25	IL-6	Interleukin-6
CCL28	C-C motif chemokine 28	IL-7	Interleukin-7
CD40	CD40L receptor	IL-8	Interleukin-8
CDCP1	CUB domain-containing protein 1	IL10	Interleukin-10
CXCL1	C-X-C motif chemokine 1	IL-10RA	Interleukin-10 receptor subunit alpha
CXCL5	C-X-C motif chemokine 5	IL-10RB	Interleukin-10 receptor subunit beta
CXCL6	C-X-C motif chemokine 6	IL-12B	Interleukin-12 subunit beta
CXCL9	C-X-C motif chemokine 9	IL-13	Interleukin-13
CXCL10	C-X-C motif chemokine 10	IL-15RA	Interleukin-15 receptor subunit alpha
CXCL11	C-X-C motif chemokine 11	IL-17A	Interleukin-17A
CST5	Cystatin D	IL-17C	Interleukin-17C
DNER	Delta and Notch-like epidermal growth factor-related receptor	IL-18	Interleukin-18
CCL11	Eotaxin	IL-18R1	Interleukin-18 receptor 1
4EBP1	Eukaryotic translation initiation factor 4E-binding protein 1	IL-20	Interleukin-20
FGF-21	Fibroblast growth factor 21	IL-20RA	Interleukin-20 receptor subunit alpha
FGF-23	Fibroblast growth factor 23	IL-22 RA1	Interleukin-22 receptor subunit alpha-1
FGF-5	Fibroblast growth factor 5	IL-24	Interleukin-24
FGF-19	Fibroblast growth factor 19	IL-33	Interleukin-33

Abbreviation	Name	Abbreviation	Name
LAP TGF beta-1	Latency-associated peptide transforming growth factor beta-1	SIRT2	SIR2-like protein 2
LIF	Leukemia inhibitory factor	STAMBP	STAM-binding protein
LIF-R	Leukemia inhibitory factor receptor	SCF	Stem cell factor
CSF-1	Macrophage colony-stimulating factor 1	ST1A1	Sulfotransferase 1A1
MMP-1	Matrix metalloproteinase-1	CD6	T-cell surface glycoprotein CD6 isoform
MMP-10	Matrix metalloproteinase-10	CD5	T-cell surface glycoprotein CD5
MCP-1	Monocyte chemotactic protein 1	CD8A	T-cell surface glycoprotein CD8 alpha chain
MCP-2	Monocyte chemotactic protein 2	TSLP	Thymic stromal lymphopoietin
MCP-3	Monocyte chemotactic protein 3	TNFB	TNF-beta
MCP-4	Monocyte chemotactic protein 4	TRANCE	TNF-related activation-induced cytokine
CD244	Natural killer cell receptor 2B4	TRAIL	TNF-related apoptosis-inducing ligand
NT-3	Neurotrophin-3	TGF-alpha	Transforming growth factor alpha
NRTN	Neurturin	TWEAK	Tumor necrosis factor (ligand) superfamily, member 12
OSM	Oncostatin-M	TNF	Tumor necrosis factor
OPG	Osteoprotegerin	TNFSF14	Tumor necrosis factor ligand superfamiliy member 14
PD-L1	Programmed cell death 1 ligand 1	TNFRSF9	Tumor necrosis factor receptor superfamily member 9
EN-RAGE	Protein S100-A12	uPA	Urokinase-type plasminogen activator
SLAMF1	Signalling lymphocytic activation molecule	VEGF-A	Vascular endothelial growth factor A
**Biomarkers excluded from analyses**			
Beta-NGF	Beta-nerve growth factor	IL-1 alpha	Interleukin-1-alpha
IL-2	Interleukin-2	IL-4	Interleukin-4
IL-5	Interleukin-5	IL-13	Interleukin-13
IL-20	Interleukin-20	IL-24	Interleukin-24
IL-33	Interleukin-33	LIF	Leukemia inhibitory factor
TSLP	Thymic stromal lymphopoietin		

Supplementary table 2

Main effect of sex on protein abundance in the total population, adjusted for obesity status, age, smoking status, diabetes mellitus, hypertension, and eGFR. The Benjamini–Hochberg correction was applied to account for multiple testing across the entire panel.

	Beta (men vs women)	SE	Nominal p	N	q_BH	Direction
CX3CL1	-0.8481	0.1537	6.23e-08	403	5.05e-06	Higher in women
CSF_1	-0.3572	0.0722	1.13e-06	403	4.57e-05	Higher in women
CD5	-0.4923	0.1098	9.61e-06	403	0.00026	Higher in women
FGF_23	-0.5021	0.1295	0.000123	403	0.00199	Higher in women
TGF_alpha	-0.7102	0.1831	0.000123	403	0.00199	Higher in women
uPA	-0.3604	0.0951	0.000175	403	0.00237	Higher in women
TNFRSF9	-0.4307	0.1162	0.00024	403	0.00278	Higher in women
CCL23	-0.4481	0.1342	0.000917	403	0.00834	Higher in women
SCF	-0.4408	0.1321	0.000927	403	0.00834	Higher in women
CXCL6	-0.5944	0.1877	0.00166	403	0.0135	Higher in women
IL_15RA	-0.3111	0.1062	0.0036	403	0.0265	Higher in women
CD244	-0.3101	0.1105	0.00526	403	0.0355	Higher in women
CST5	-0.4402	0.1614	0.00666	403	0.0415	Higher in women
LIF_R	-0.2386	0.0937	0.0113	403	0.0608	Higher in women
CXCL10	-0.6119	0.2442	0.0126	403	0.0608	Higher in women
IL_10RB	-0.2563	0.1026	0.0129	403	0.0608	Higher in women
CD6	-0.4466	0.1793	0.0131	403	0.0608	Higher in women
IL_12B	-0.5301	0.2149	0.0141	403	0.0608	Higher in women
IL10	-0.5537	0.2249	0.0143	403	0.0608	Higher in women
CCL25	-0.5058	0.2131	0.0181	403	0.0734	Higher in women
TWEAK	-0.249	0.1063	0.0197	403	0.0759	Higher in women
CD8A	-0.4693	0.2225	0.0355	403	0.131	Higher in women
NT_3	-0.3509	0.1727	0.0429	403	0.151	Higher in women
ADA	-0.242	0.1205	0.0453	403	0.153	Higher in women
PD_L1	-0.285	0.144	0.0485	403	0.157	Higher in women
CXCL1	-0.3317	0.1746	0.0582	403	0.181	Higher in women
TNFSF14	-0.3593	0.1922	0.0623	403	0.187	Higher in women
IL_17C	-0.4694	0.2552	0.0666	403	0.192	Higher in women
CD40	-0.1789	0.098	0.0687	403	0.192	Higher in women
OSM	-0.4435	0.2473	0.0736	403	0.195	Higher in women
NRTN	0.4282	0.2396	0.0747	403	0.195	Higher in men
HGF	-0.2033	0.1207	0.093	403	0.229	Higher in women
FGF_5	-0.2213	0.1316	0.0935	403	0.229	Higher in women
TRANCE	-0.3531	0.2206	0.11	403	0.263	Higher in women
DNER	0.1266	0.0829	0.128	403	0.295	Higher in men
CCL19	-0.3523	0.2379	0.14	403	0.314	Higher in women
GDNF	-0.1949	0.1353	0.15	403	0.329	Higher in women

OPG	-0.1503	0.1072	0.162	403	0.34	Higher in women
Flt3L	-0.1871	0.1341	0.164	403	0.34	Higher in women
IL_20RA	-0.2748	0.2184	0.209	403	0.417	Higher in women
TNFB	-0.189	0.1509	0.211	403	0.417	Higher in women
CCL3	-0.2394	0.195	0.22	403	0.425	Higher in women
CXCL9	-0.319	0.2658	0.231	403	0.432	Higher in women
CCL28	-0.2102	0.1767	0.235	403	0.432	Higher in women
CCL11	0.1454	0.1236	0.24	403	0.432	Higher in men
4E_BP1	0.4093	0.3573	0.253	403	0.445	Higher in men
MMP_10	-0.2336	0.2141	0.276	403	0.476	Higher in women
SLAMF1	-0.1654	0.1567	0.292	403	0.493	Higher in women
MCP_1	-0.1511	0.1515	0.319	403	0.528	Higher in women
IL_2RB	-0.1604	0.1719	0.351	403	0.569	Higher in women
CXCL11	-0.2224	0.2475	0.37	403	0.587	Higher in women
EN_RAGE	-0.2355	0.2679	0.38	403	0.591	Higher in women
CASP_8	-0.0971	0.1129	0.39	403	0.591	Higher in women
MCP_2	0.1819	0.2131	0.394	403	0.591	Higher in men
FGF_19	0.2627	0.325	0.419	403	0.618	Higher in men
TRAIL	-0.0737	0.1011	0.467	403	0.675	Higher in women
MCP_3	-0.1358	0.196	0.489	403	0.689	Higher in women
CXCL5	-0.1268	0.185	0.494	403	0.689	Higher in women
CCL4	-0.1271	0.2019	0.529	403	0.722	Higher in women
VEGFA	-0.1263	0.2033	0.535	403	0.722	Higher in women
ST1A1	0.1628	0.2734	0.552	403	0.733	Higher in men
SIRT2	0.0995	0.1744	0.569	403	0.743	Higher in men
MMP_1	0.1242	0.2629	0.637	403	0.819	Higher in men
ARTN	0.0779	0.1803	0.666	403	0.832	Higher in men
IL_10RA	0.1276	0.2968	0.668	403	0.832	Higher in men
STAMBP	-0.0529	0.1311	0.687	403	0.843	Higher in women
IL_22_RA1	-0.0927	0.2458	0.706	403	0.854	Higher in women
IL8	0.0631	0.1792	0.725	403	0.861	Higher in men
AXIN1	-0.0471	0.1382	0.734	403	0.861	Higher in women
MCP_4	-0.0582	0.1876	0.756	403	0.875	Higher in women
LAP_TGF_beta_1	-0.0415	0.1664	0.803	403	0.906	Higher in women
CCL20	-0.0816	0.3311	0.805	403	0.906	Higher in women
CDCP1	-0.0397	0.1819	0.827	403	0.91	Higher in women
FGF_21	0.0957	0.4483	0.831	403	0.91	Higher in men
IL7	-0.0261	0.1394	0.852	403	0.92	Higher in women
IFN_gamma	-0.0582	0.368	0.874	403	0.932	Higher in women
IL_18R1	-0.018	0.1405	0.898	403	0.945	Higher in women
TNF	0.0169	0.1776	0.924	403	0.953	Higher in men
IL18	0.0153	0.1734	0.93	403	0.953	Higher in men
IL6	-0.0175	0.2826	0.951	403	0.961	Higher in women
IL_17A	-0.0109	0.2206	0.961	403	0.961	Higher in women
